# Genetic inference of immune responsiveness of melanoma cancer patients to TIL therapy

**DOI:** 10.1186/2051-1426-1-S1-P63

**Published:** 2013-11-07

**Authors:** Michele Sommariva, Foo Cheung, Jinguo Chen, Rongye Shi, Ling Zhang, Richard Morgan, Davide Bedognetti, Valeria De Giorgi, Maria L Ascierto, Steven A Rosenberg, Francesco M Marincola, Ena Wang

**Affiliations:** 1 Infectious Disease and Immunogenetics Section (IDIS), Department of Transfusion Medicine, Clinical Center and trans-NIH Center for Human Immunology (CHI), National Institutes of Health, Bethesda, MD, USA; 2 Department of Biomedical Sciences for Health, University of Milan, Milan, Italy; 3 Surgery Branch, National Cancer Institute, Bethesda, MD, USA; 4 Sidra Medical and Research Center, Doha, Qatar

## 

Adoptive cell transfer therapy (ACT) holds a highly promising treatment for metastatic melanoma patients. ACT involves the ex vivo expansion of autologous antitumor reactive tumor infiltrating lymphocytes and their reinfusion into lymphodepleted patients, accompanied by IL-2 administration. ACT demonstrates objective clinical responsiveness in 40-50% patients, including 10-21% complete responses. However, the mechanisms underlying the observed inter-individual variability in response to this type of therapy are not well understood. We applied whole genome-wide association study (GWAS) using Illumina OmniQuad 1M SNP bead array and expanded the SNP coverage to 33M by imputation against International HapMap and 1,000 genomes data sets. The analysis was performed in 208 TIL or PBMC samples undergone adoptive therapy or high does IL-2 therapy at the Surgery Branch, NCI. Comparisons between patients experiencing a complete responder (CR, n=30) versus those suffering progression of disease (PD, n=108) excluding the reminder partial responder patients identified 140 independent association SNPs which covers 14 functional known genes at a threshold p-value < 1 x 10^-5^. Within the chromosomal crossover range (1000Kb), we observed possible association with 405 annotated genes. The strongest signal was rs11587096, rs4506458 on chromosome 1 and rs7894773, rs7070986 at chromosome 10. Several genes at those loci had known immunological function. Those include TLR5, MIA3, TAF1A, DUSP10, DISP1, HHIPL2, MAF177B and EIF3a, FAM45A, SFXN4, GRK5, RGS10, TIAL1, BAG3, INPPF5 AND SEC23IP on chromosome 1 and 10 respectively. Possible functional impact of the significant SNP on chromosome 10 at transcript level was further validated by analyzing encoded gene exons using Fluidigm technology. In conclusion, we have identified different loci associated with clinic outcome by GWAS study suggesting that genetic predisposition is one of the important component determine immune responsiveness.

